# Supplementary material for: Tuning nonlinear damping in graphene nanoresonators by parametric–direct internal resonance
Source: Nat Commun. 2021 Feb 17;12:1099. doi: 10.1038/s41467-021-21334-w (PMC7889630; doi:10.1038/s41467-021-21334-w)
Supplement: Supplementary file 1 — Supplementary Information [file 41467_2021_21334_MOESM1_ESM.pdf]

Supplementary Information: Tuning nonlinear damping in graphene nanoresonators by parametric-direct internal resonance

Ata Keşkekler,[1] Oriel Shoshani,[2] Martin Lee,[3] Herre S. J. van der Zant,[3] Peter G. Steeneken,[1,3] and Farbod Alijani[1]

[1] *Department of Precision and Microsystems Engineering, TU Delft, The Netherlands*
[2] *Department of Mechanical Engineering, Ben-Gurion University of Negev, Israel*
[3] *Kavli Institute of Nanoscience, TU Delft, The Netherlands*

PACS numbers:

SUPPLEMENTARY NOTE I. MODEL FITS USING THE SINGLE MODE EQUATION

In this section, we use Eq. (1) to fit the experimental data. The fittings are carried out by using an algorithm that compares measurements to the analytical solution [1] to sequentially extract the model parameters. To fit the data, information about resonance frequency ω_1 and damping coefficient τ_1 are directly extracted from the measurements. The linear quality factor Q of the resonator is determined at low drive level to be 454 and has been fixed throughout the fitting procedure. Moreover, the magnitude of the parametric drive F_1 at low driving powers is estimated by tracing the locus of the pitch-fork bifurcation points (Mathieu tongue) as shown in Supplementary Figure 1. However, for higher power levels, the bifurcation points did not give reliable information due to frequency fluctuations. Thus, to obtain the parametric forces at high drive powers, we use lower force levels (known from Mathieu tongue) and extrapolate using the ratios between the applied voltage/power levels from the VNA and the forces such that $F_{1,\mathrm{high}} = F_{1,\mathrm{low}} \frac{P_{\mathrm{high}}}{P_{\mathrm{low}}}$. In other words, we assume that the drive levels applied in the experiments using the VNA are linearly related to the force felt by the resonator.

Once F_1 is estimated, γ is fitted by minimizing the curvatures of the hardening type nonlinearity observed in the experiments and the model. Finally, to match the peak amplitudes observed in the experiments, the nonlinear damping coefficient τ_{nl1} is used as the fitting parameter such that the saddle-node bifurcation amplitude (A_{SNB}) and frequency (f_{SNB}) are within 0.1% error of the A_{SNB} and f_{SNB} obtained experimentally. In Supplementary Figure 2 we report the fitted curves and their associated γ and τ_{nl1}. Fits are associated with the experimental curves(Fig. 1c) that are repeated with labels as Supplementary Figure 3 for convinience.

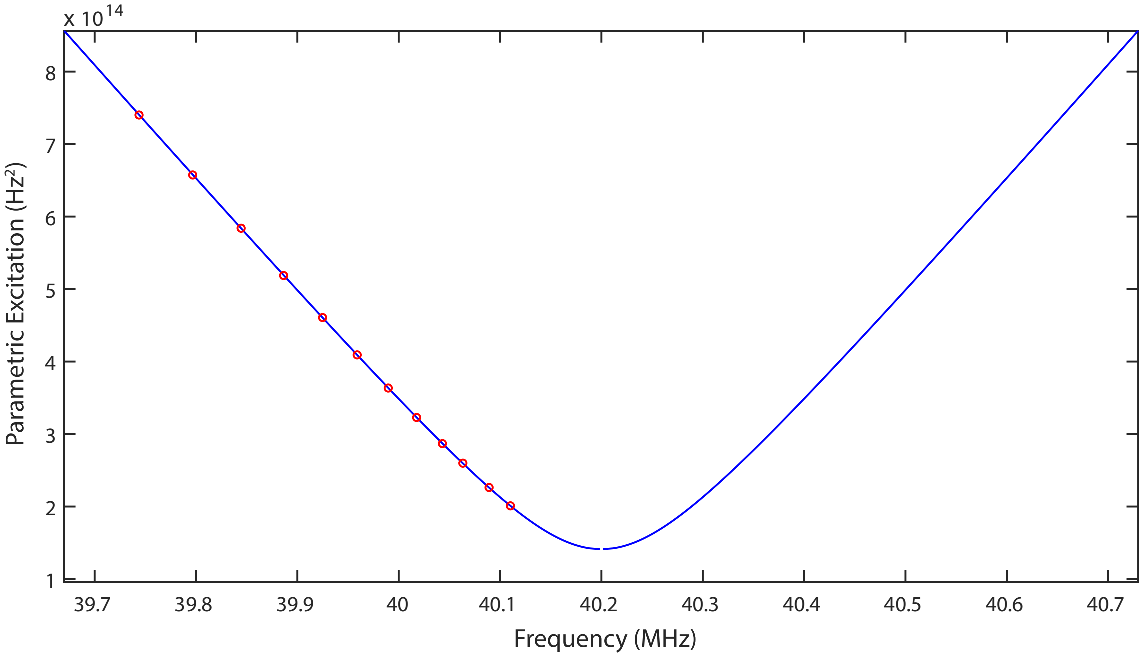

Supplementary Figure 1: **Determination of parametric excitation levels.** The Mathieu tongue(blue) associated with the experimental pitch-fork bifurcation frequencies is used to extract parametric excitation levels(red).

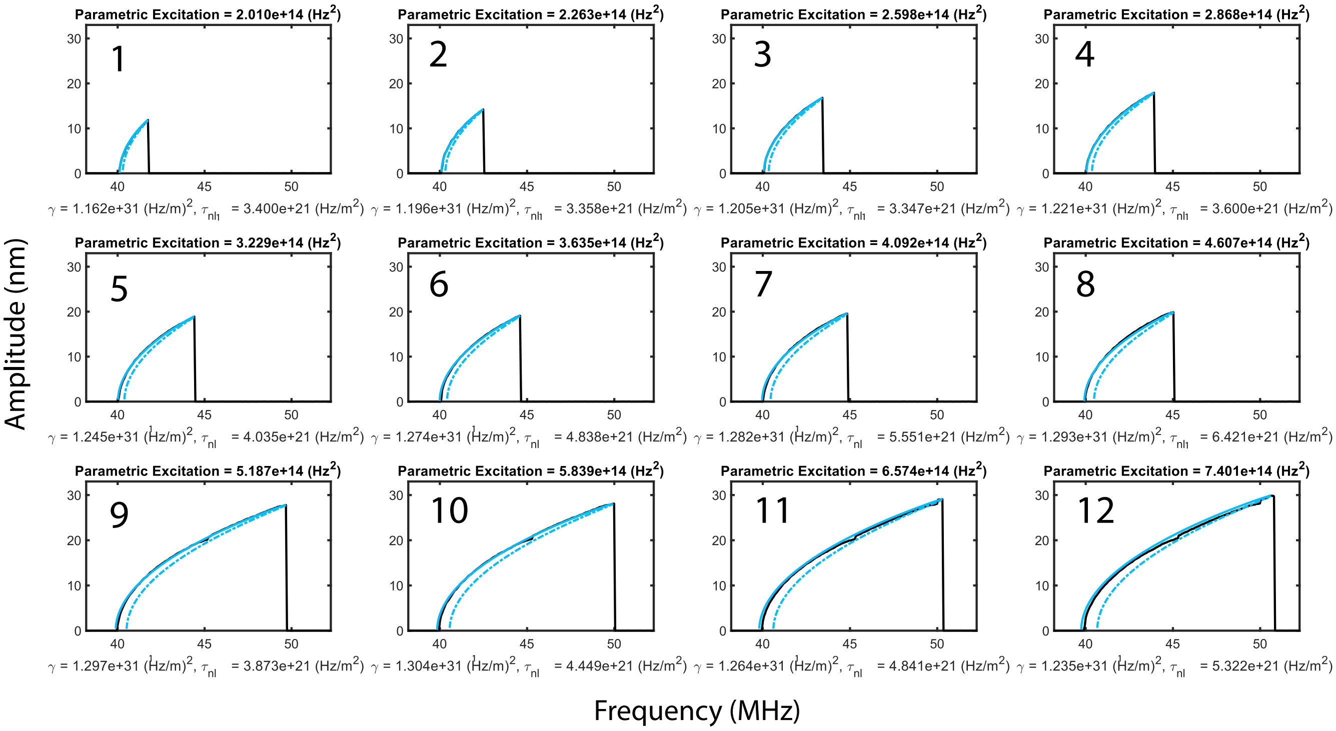

Supplementary Figure 2: **Single mode model fits.** Frequency response measurements(black) and corresponding fits(blue) obtained by using the single mode model. The solid and dashed lines in the figure represent stable and unstable numerical solutions, respectively.

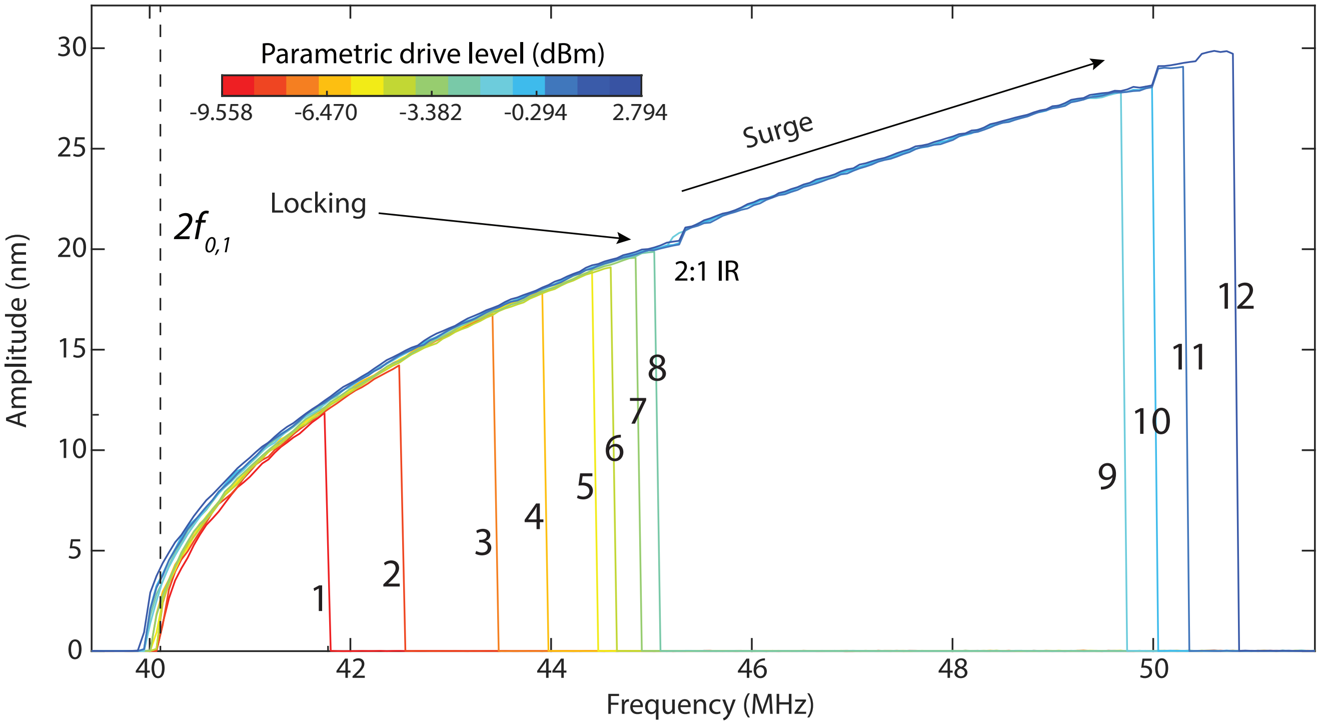

Supplementary Figure 3: **Experimental parametric resonance curves.** Experimental frequency response curves from the main text Fig. 1c, labelled to corresponding fitted curves in Supplementary Figure 2

SUPPLEMENTARY NOTE II. EXPERIMENT ON A SECOND NANODRUM

Here we present additional experimental evidence of a parametric-direct internal resonance in a second graphene nanodrum that is 14 nm thick and has a radius of 2.5 μm. Supplementary Figure 4a shows the direct response and Supplementary Figure 4b the parametric resonance where the internal resonance frequency f_{IR} occurs close to 2.3 times the fundamental mode, and after which an amplitude-frequency surge is apparent.

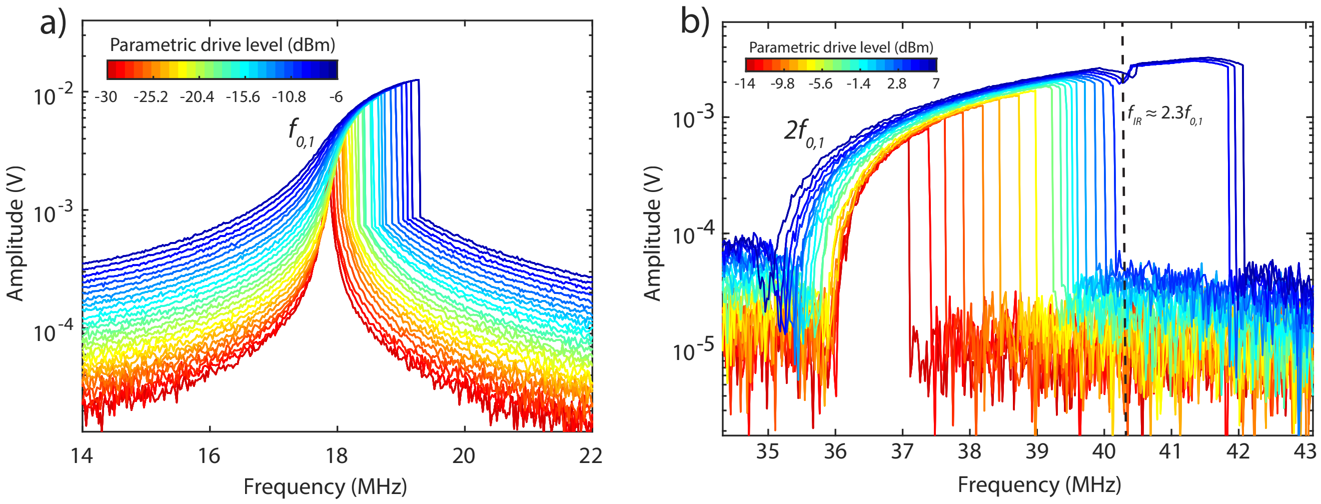

Supplementary Figure 4: **Frequency response measurements.** Frequency response of (a) direct and (b) parametric resonance in a second graphene nanodrum, showing the parametric-direct interaction.

SUPPLEMENTARY NOTE III. NORMAL FORM OF THE EQUATIONS OF MOTION

In the absence of damping and external forces, the geometrically nonlinear equations of motion for plates and membranes can be written in the following discretized form [2]

$$\ddot{x}_k + \omega_k^2 x_k + \sum_n \sum_p \alpha_{np}^{(k)} x_n x_p + \sum_n \sum_p \sum_l \gamma_{npl}^{(k)} x_n x_p x_l = 0, \quad \forall k \in \mathbb{N}, \tag{1}$$

where ω_k is the eigenfrequency of the kth mode x_k, and $\alpha_{np}^{(k)}$ and $\gamma_{npl}^{(k)}$ are quadratic and cubic nonlinear terms, respectively. For a two mode system ($k = 2$), the nonlinear equations become

$$\ddot{x}_1 + \omega_1^2 x_1 + \alpha_{11}^{(1)} x_1^2 + \alpha_{12}^{(1)} x_1 x_2 + \alpha_{22}^{(1)} x_2^2 + \gamma_{111}^{(1)} x_1^3 + \gamma_{112}^{(1)} x_1^2 x_2 + \gamma_{122}^{(1)} x_1 x_2^2 + \gamma_{222}^{(1)} x_2^3 = 0, \tag{2}$$

$$\ddot{x}_2 + \omega_2^2 x_2 + \alpha_{11}^{(2)} x_1^2 + \alpha_{12}^{(2)} x_1 x_2 + \alpha_{22}^{(2)} x_2^2 + \gamma_{111}^{(2)} x_1^3 + \gamma_{112}^{(2)} x_1^2 x_2 + \gamma_{122}^{(2)} x_1 x_2^2 + \gamma_{222}^{(2)} x_2^3 = 0. \tag{3}$$

Note that many of the nonlinear terms in Supplementary Equation (2) and (3) are non-resonant, and hence, can be eliminated via a normal form transformation [3]. To recover the resonant terms (which cannot be eliminated from the normal form) in a 2:1 internal resonance condition ($\omega_2 \simeq 2\omega_1$), here instead we assume harmonic motion of the form $x_1 \approx \cos(\omega_1 t)$ and $x_2 \approx \cos(2\omega_1 t)$ as a first approximation. Inserting these relations in Supplementary Equation (2) reveals that the terms $x_1^3 \approx \frac{3}{4}\cos(\omega_1 t) + \frac{1}{4}\cos(3\omega_1 t)$ and $x_1 x_2 \approx \frac{1}{2}(\cos(\omega_1 t) + \cos(3\omega_1 t))$ in the first equation of motion are trivially resonant. The same holds for the term $x_1^2 \approx= \frac{1}{2}(1+\cos(2\omega_1 t))$, which can be viewed as a resonant term for Supplementary Equation (3). Furthermore, in order to obtain the most simple model for the considered dynamical system, we neglect the contribution of the dispersive coupling terms $x_1 x_2^2$ in Supplementary Equation (2) and $x_2^2 x_1$ in Supplementary Equation (3) (which only shift the resonance frequency of each mode in amount that is proportional to the amplitude square of the other mode), and the Duffing nonlinearity of the second mode (which is assumed to operate below the Duffing threshold). Therefore, the governing equations of motion reduce to

$$\ddot{x}_1 + \omega_1^2 x_1 + \alpha_{12}^{(1)} x_1 x_2 + \gamma_{111}^{(1)} x_1^3 = 0, \tag{4}$$

$$\ddot{x}_2 + \omega_2^2 x_2 + \alpha_{11}^{(2)} x_1^2 = 0. \tag{5}$$

We note that Supplementary Equation (4)-(5) are the normal form of the conservative dynamical system of interest, with $\gamma_{111}^{(1)}{=}\gamma$ being the Duffing nonlinearity of the first mode, and $\alpha{=}\alpha_{12}^{(1)}/2{=}\alpha_{11}^{(2)}$ is the coupling coefficient, which stems from a single-term potential $U_{\rm cp} = \alpha x_1^2 x_2$.

SUPPLEMENTARY NOTE IV. SLOW DYNAMIC EQUATIONS AND BIFURCATION ANALYSIS

In order to investigate the experimentally observed physics in our graphene nanodrum, we use the normal form of the equations obtained in the previous section in the presence of damping and external forcing terms. The resulting equations then read

$$\ddot{x}_1 + \omega_1^2 x_1 + \gamma x_1^3 + 2\alpha x_2 x_1 = F_1 x_1 \cos(\omega_F t) - 2\tau_1 \dot{x}_1 - 2\tau_{nl1} x_1^2 \dot{x}_1, \tag{6}$$

$$\ddot{x}_2 + \omega_2^2 x_2 + \alpha x_1^2 = F_2 \cos(\omega_F t) - 2\tau_2 \dot{x}_2, \tag{7}$$

in which τ_1 and τ_2 are the damping coefficients associated with modes 1 and 2 , respectively. F_1 is the parametric drive, F_2 is the direct drive, and τ_{nl1} is the van der Pol type nonlinear damping term added to the equation of motion to avoid unbounded parametric resonance [1].

Applying the rotating wave approximation (RWA) $x_1(t) = A_1(t)\exp(i\omega_F t/2) + A_1^*(t)\exp(-i\omega_F t/2)$, $\dot{x}_1(t) = (i\omega_F/2)[A_1(t)\exp(i\omega_F t/2) - A_1^*(t)\exp(-i\omega_F t/2)]$, $x_2 = A_2(t)\exp(i\omega_F t) + A_2^*(t)\exp(-i\omega_F t)$, $\dot{x}_2 = i\omega_F[A_2(t)\exp(i\omega_F t) - A_2^*(t)\exp(-i\omega_F t)]$, where A_j and A_j^* are the complex-amplitude of the jth mode and its complex-conjugate, respectively, and introducing the detuning parameters $\Delta\omega_1 = \omega_F/2 - \omega_1$, $\Delta\omega_2 = \omega_F - \omega_2$, we obtain the following evolution equations for the complex amplitudes

$$\dot{A}_1 = -\left[\tau_1 + \tau_{nl1}|A_1|^2 + i\left(\Delta\omega_1 - \frac{3\gamma}{\omega_F}|A_1|^2\right)\right]A_1 + \frac{2i}{\omega_F}\left(\alpha A_2 - \frac{F_1}{4}\right)A_1^*, \tag{8}$$

$$\dot{A}_2 = -(\tau_2 + i\Delta\omega_2)A_2 + \frac{i}{2\omega_F}\left(\alpha A_1^2 - \frac{F_2}{2}\right). \tag{9}$$

At steady-state $\dot{A}_j = 0$. Thus, from Supplementary Equation (9) we find that the steady-state complex-amplitude of the second mode A_{2ss} is given by

$$A_{2ss} = \frac{(i\tau_2 + \Delta\omega_2)}{2\omega_F(\tau_2^2 + \Delta\omega_2^2)}\left(\alpha A_{1ss}^2 - \frac{F_2}{2}\right). \tag{10}$$

Hence, by substituting Supplementary Equation (10) into Supplementary Equation (8), we obtain a single equation for the steady-state complex-amplitude of the first mode A_{1ss} as follows

$$\left[\tau_1 + \left(\tau_{nl1} + \frac{\alpha^2\tau_2}{\omega_F^2(\tau_2^2 + \Delta\omega_2^2)}\right)|A_{1ss}|^2\right]A_{1ss} + i\left[\Delta\omega_1 - \left(\frac{3\gamma}{\omega_F} + \frac{\alpha^2\Delta\omega_2}{\omega_F^2(\tau_2^2 + \Delta\omega_2^2)}\right)|A_{1ss}|^2\right]A_{1ss}$$
$$= -\frac{i}{2\omega_F}\left(F_1 + \frac{\alpha(i\tau_2 + \Delta\omega_2)}{\omega_F(\tau_2^2 + \Delta\omega_2^2)}F_2\right)A_{1ss}^*. \tag{11}$$

Taking the magnitude squared of both sides of Supplementary Equation (11), we find the intensity of the non-trivial response of the first mode $4|A_{1ss}|^2 \equiv a_1^2$ as the positive roots of the equation

$$\left[\tau_1 + \left(\tau_{nl1} + \frac{\alpha^2\tau_2}{\omega_F^2(\tau_2^2 + \Delta\omega_2^2)}\right)\frac{a_1^2}{4}\right]^2 + \left[\Delta\omega_1 - \left(\frac{3\gamma}{\omega_F} + \frac{\alpha^2\Delta\omega_2}{\omega_F^2(\tau_2^2 + \Delta\omega_2^2)}\right)\frac{a_1^2}{4}\right]^2$$
$$= \frac{1}{4\omega_F^2}\left[F_1^2 + \frac{\alpha F_2(\alpha F_2 + 2\omega_F\Delta\omega_2 F_1)}{\omega_F^2(\tau_2^2 + \Delta\omega_2^2)}\right]. \tag{12}$$

We note that Supplementary Equation (12) is a quadratic equation in a_1^2. Thus, in addition to the trivial solution $a_1 = 0$, which is always a solution, we are left with, at most, two additional positive solutions for a_1. Therefore,

the non-trivial steady-state solutions of the first mode $x_1 = a_1\cos(\omega_F t/2+\phi_1)$ is fully described by Supplementary Equation (12) and the following equation for the phase

$$e^{2i\phi_1} = \frac{A_{1ss}}{A^*_{1ss}} = \frac{-\frac{i}{2\omega_F}\left(F_1 + \frac{\alpha(i\tau_2+\Delta\omega_2)}{\omega_F(\tau_2^2+\Delta\omega_2^2)}F_2\right)}{\tau_1 + \left(\tau_{nl1} + \frac{\alpha^2\tau_2}{\omega_F^2(\tau_2^2+\Delta\omega_2^2)}\right)\frac{a_1^2}{4} + i\left[\Delta\omega_1 - \left(\frac{3\gamma}{\omega_F} + \frac{\alpha^2\Delta\omega_2}{\omega_F^2(\tau_2^2+\Delta\omega_2^2)}\right)\frac{a_1^2}{4}\right]}. \tag{13}$$

From Supplementary Equation (10) we also find the steady-state solution of the second mode $A_{2ss} = a_2 e^{i\phi_2}/2$. Thus, the solution a_1 of Supplementary Equation (12) along with Supplementary Equations (10), (13) give complete description of the system non-trivial steady-state solutions. Note that the peak amplitude of the first mode A_{SNB} can be calculated by differentiation of Supplementary Equation (12) with respect to ω_F and setting $da_1^2/d\omega_F = 0$. To the leading order approximation, the expression for the peak amplitude A_{SNB} is given by

$$A^2_{\mathrm{SNB}} = \frac{2\sqrt{\tau_2^2+\Delta\omega_2^2}\left[\omega_F^2(\tau_2^2+\Delta\omega_2^2)F_1^2 + \alpha F_2(\alpha F_2 + 2\omega_F\Delta\omega_2 F_1)\right]^{1/2} - 4\omega_F^2(\tau_2^2+\Delta\omega_2^2)\tau_1}{\omega_F^2(\tau_2^2+\Delta\omega_2^2)\tau_{nl1} + \alpha^2\tau_2}. \tag{14}$$

Instability threshold of the trivial solution

In order to find the conditions under which the trivial solution of the first mode $a_1 = 0$ loses its stability, we linearize Supplementary Equations (8)-(9) around $A_{1ss} = 0,\ A_{2ss} = -\frac{(i\tau_2+\Delta\omega_2)F_2}{4\omega_F(\tau_2^2+\Delta\omega_2^2)}$, and obtain the eigenvalues of the resulting linear system

$$\delta\dot{A}_1 = -\left[\tau_1 + i\Delta\omega_1\right]\delta A_1 - \frac{i}{2\omega_F}\left(F_1 + \frac{\alpha(i\tau_2+\Delta\omega_2)}{\omega_F(\tau_2^2+\Delta\omega_2^2)}F_2\right)\delta\bar{A}_1, \tag{15}$$

$$\delta\dot{A}_2 = -(\tau_2 + i\Delta\omega_2)\delta A_2. \tag{16}$$

Note that Supplementary Equation (15)-(16) are uncoupled, and hence, we can analyze each equation separately. Supplementary Equation (16) can be readily solved to yield $\delta A_2 = \delta A_2(0)e^{-(\tau_2+i\Delta\omega_2)t}$. Consequently, δA_2 decays to zero for all $\tau_2 > 0$. Similarly, we seek a solution for Supplementary Equation (15) in the form $\delta A_1 = |\delta A_1|e^{i\delta\phi_1}e^{\lambda t}$, where $\delta\phi_1$ and λ are real. By substitution of the solution into Supplementary Equation (15) we find that

$$\lambda = -\tau_1 \pm \sqrt{\frac{1}{4\omega_F^2}\left[F_1^2 + \frac{\alpha F_2(\alpha F_2 + 2\omega_F\Delta\omega_2 F_1)}{\omega_F^2(\tau_2^2+\Delta\omega_2^2)}\right] - \Delta\omega_1^2}. \tag{17}$$

Thus, for $\lambda > 0$, the trivial solution of the first mode $a_1 = 0$ is unstable (i.e., there is a supercritical pitchfork bifurcation). The boundaries, which define the domains of the instability, form the Mathieu tongue and are given by

$$4\omega_F^2(\tau_1^2+\Delta\omega_1^2) = F_1^2 + \frac{\alpha F_2(\alpha F_2 + 2\omega_F\Delta\omega_2 F_1)}{\omega_F^2(\tau_2^2+\Delta\omega_2^2)}. \tag{18}$$

Note that the same condition can be obtained from Supplementary Equation (12) by taking the limit $a_1 \to 0$.

Stability of the non-trivial steady-state solutions and local bifurcation analysis

To investigate the stability of the non-trivial steady-state solutions [which are found from Supplementary Equation (10) and Supplementary Equations (12)-(13)], we superimpose a perturbation $\delta\mathbf{u} = (\delta A_1, \delta A_2)^T$ on the non-trivial fixed-points of Supplementary Equations (8)-(9) $\mathbf{u}_{ss} = (A_{1ss}, A_{2ss})^T$, linearize in terms of the perturbed variables, and obtain the following pair of linear complex evolution-equations for the perturbation $\delta\mathbf{u}$

$$\begin{aligned}\delta\dot{A}_1 = &-\left[\tau_1 + 2\tau_{nl1}|A_{1ss}|^2 + i\left(\Delta\omega_1 - \frac{6\gamma}{\omega_F}|A_{1ss}|^2\right)\right]\delta A_1 \\ &-\left[\tau_{nl1}A_{1ss}^2 + \frac{i}{\omega_F}\left(\frac{F_1}{2} - 2\alpha A_{2ss} - 3\gamma A_{1ss}^2\right)\right]\delta\bar{A}_1 + \frac{2i\alpha}{\omega_F}\bar{A}_{1ss}\delta A_2,\end{aligned} \tag{19}$$

$$\delta\dot{A}_2 = \frac{i\alpha}{\omega_F}A_{1ss}\delta A_1 - (\tau_2 + i\Delta\omega_2)\delta A_2. \tag{20}$$

Using Cartesian notations for the perturbations $\delta A_j = q_j + ip_j$, Supplementary Equations (19)-(20) can be written as $\dot{q}_1 + i\dot{p}_1 = f_1 + if_2$ and $\dot{q}_2 + i\dot{p}_2 = f_3 + if_4$. Thus, by taking the real and imaginary part of these equations, we obtain a

set of four linear real evolution-equations for the perturbation quadratures $\dot{\boldsymbol{\eta}} = \mathbf{J}\cdot\boldsymbol{\eta}$, where $\boldsymbol{\eta} = (q_1, p_1, q_2, p_2)^T$, $J_{nm} = \partial f_n/\partial\eta_m|_{A_{1ss},A_{2ss}}$. Therefore, the stability of the fixed points are determined by the eigenvalues λ of $\mathbf{J}$, which are the roots of the following characteristic polynomial

$$\lambda^4 + c_1\lambda^3 + c_2\lambda^2 + c_3\lambda + c_4 = 0$$
$$c_1 = -\mathrm{tr}(\mathbf{J}),\ c_2 = \frac{1}{2}(\mathrm{tr}(\mathbf{J})^2 - \mathrm{tr}(\mathbf{J^2})),\ c_3 = -\frac{1}{3}\left(\mathrm{tr}(\mathbf{J^3}) - \frac{3}{2}\mathrm{tr}(\mathbf{J}^2)\mathrm{tr}(\mathbf{J}) + \mathrm{tr}(\mathbf{J})^3\right),\ c_4 = \det(\mathbf{J}). \tag{21}$$

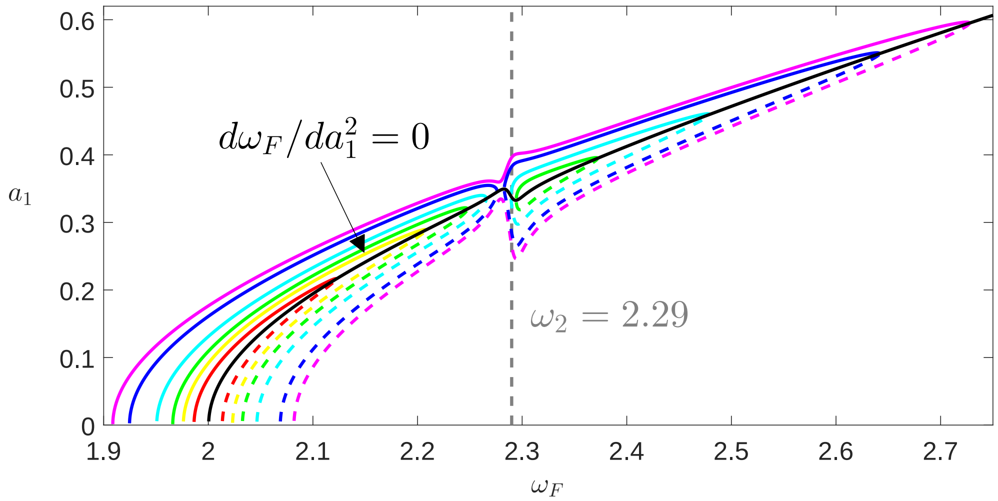

Supplementary Figure 5: **Theoretical parametric resonance curves**. The parameter values used for the curves are: $\tau_1 = 0.0025, \tau_{nl1} = 0.175, \tau_2 = 0.00764, \alpha = 0.17, \gamma = 3.7, \omega_1 = 1, \omega_2 = 2.29, F_2 = 0.13F_1$. The magenta/dark-blue/light-blue/green/yellow/red curves are the first mode response curve for drive amplitudes $F_1 = 0.18, 0.15, 0.1, 0.07, 0.05, 0.03$. The grey vertical dashed line shows the frequency of the second mode, and the black curve show the loci of the saddle-node bifurcations.

Consequently, there is a saddle-node bifurcation whenever $c_4 = 0$, and Hopf bifurcation whenever $\Delta_3 = c_3(c_2c_1 - c_3) - c_4c_1^2 = 0$. Furthermore, near the Hopf threshold ($\Delta_3 = 0$), the frequency of the limit cycle is given by $\lambda_{1,2} = \pm i\Omega_H$, $\Omega_H^2 = c_3/c_1$. Note that the condition for the saddle-node bifurcation $c_4 = 0$ can also be obtained by differentiating Supplementary Equation (12) with respect to the drive frequency ω_F, and requiring that $d\omega_F/da_1^2 = 0$ (i.e., requirement of vertical tangency in the response curve). Supplementary Figure 5 shows the response curves for several values of drive amplitude F_1 along with the locus of the saddle-node bifurcations, and an indication of the second mode frequency (assuming that ω_2=2.29 ω_1). It is clear from the figure that as the driving amplitude F_1 increases, the gap between the first two saddle-node bifurcations decreases. At a critical drive level (the magenta response curve), these two saddle-node bifurcations annihilate each other and the response becomes continuous.

SUPPLEMENTARY NOTE V. MODEL FITS USING THE COUPLED MODEL

To fit with the coupled model, Supplementary Equations (6) and (7) are used. The parameters from the single mode fits in the uncoupled regime (at the force levels where the coupling effects are negligible) have been taken as base parameters, since they are intrinsic to the modes themselves. This leaves only 2 additional parameters for fitting, namely the coupling strength α and the direct forcing F_2. Before the interaction, F_2 can be directly obtained from the measurements, however during the interaction the individual amplitude of the second mode is hidden beneath the parametric resonance curve. Thus, we assume a linear relation between F_1 and F_2 such that $F_2 = c_{pm}F_1$ so as to estimate the contribution of F_2 in the coupled mode dynamics. Finally, we use α as the fit parameter and minimize the error between the resonance peak (A_{SNB}) of the model and experimental data.

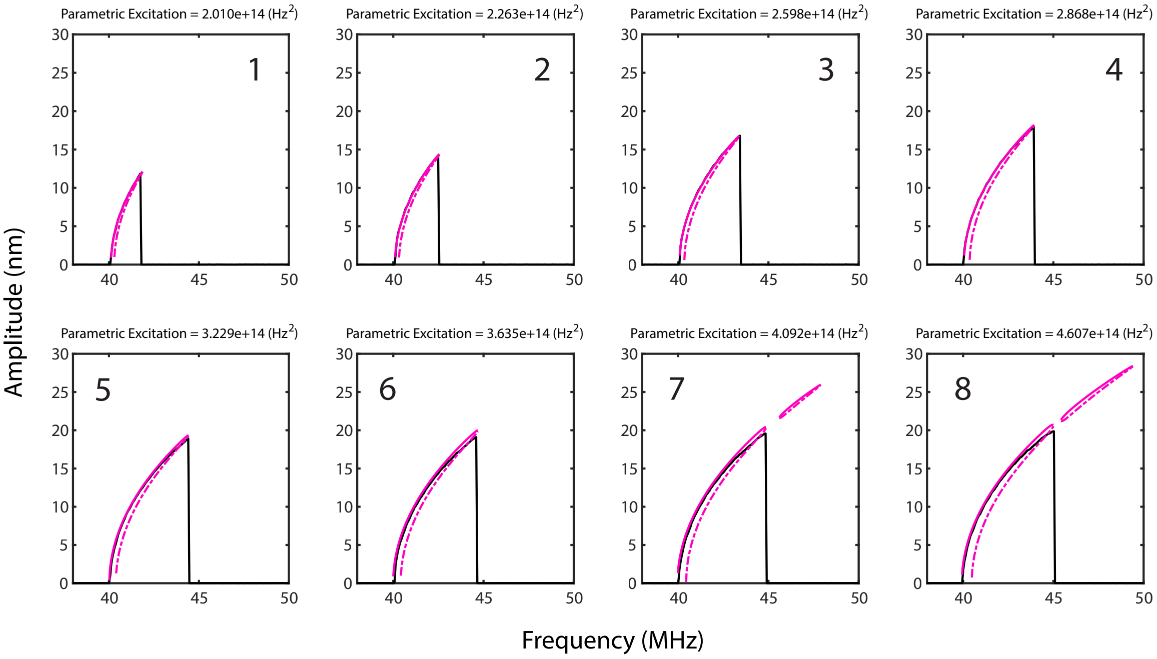

Supplementary Figure 6: **2 mode coupled model fits.** Frequency response measurements (black) and corresponding fits (pink) obtained by using the 2 mode coupled model. The solid and dashed lines in the figure represent stable and unstable numerical solutions, respectively.

In Supplementary Figure 6 we report the fits to the experimental data using the coupled model(labelled to their corresponding experimental curves in Supplementary Figure 3), and in Table I, we report the fitted values. We note that the curves with different parametric excitation are fitted with constant parameters.

TABLE I: Parameter set extracted from the fits of Supplementary Figure 6

w_1	w_2	τ_1	τ_2	τ_{nl1}	γ	α	c_{pm}
1.262×10^8 (rad/s)	2.841×10^8 (rad/s)	2.781×10^5 (Hz)	2.185×10^6 (Hz)	3.139×10^{21} (Hz/m^2)	1.205×10^{31} (Hz/m)^2	2.213×10^{22} (Hz^2/m)	1.585×10^{-9} (m)

SUPPLEMENTARY NOTE VI. EFFECT OF NONLINEAR DAMPING ON THE DIRECT RESONANCE

The effect of nonlinear damping can also be seen in the decaying responsivity of the Duffing response as the drive level increases(see Supplementary Figure 7)[1]. In order to check the consistency of the extracted nonlinear damping from parametric resonance, here we fit the direct response using the extracted intrinsic damping(τ_{nl1}) and Duffing nonlinearity(γ) reported in Table I. Supplementary Figure 8 shows reasonably good fits for different drive levels. Eq. 1 from the main text has been used as the model for these fits with a modification of the direct drive term(considering only direct excitation i.e. $F_1 x_1 cos(\omega_F t)$ has changed to $F_1 cos(\omega_F t)$).

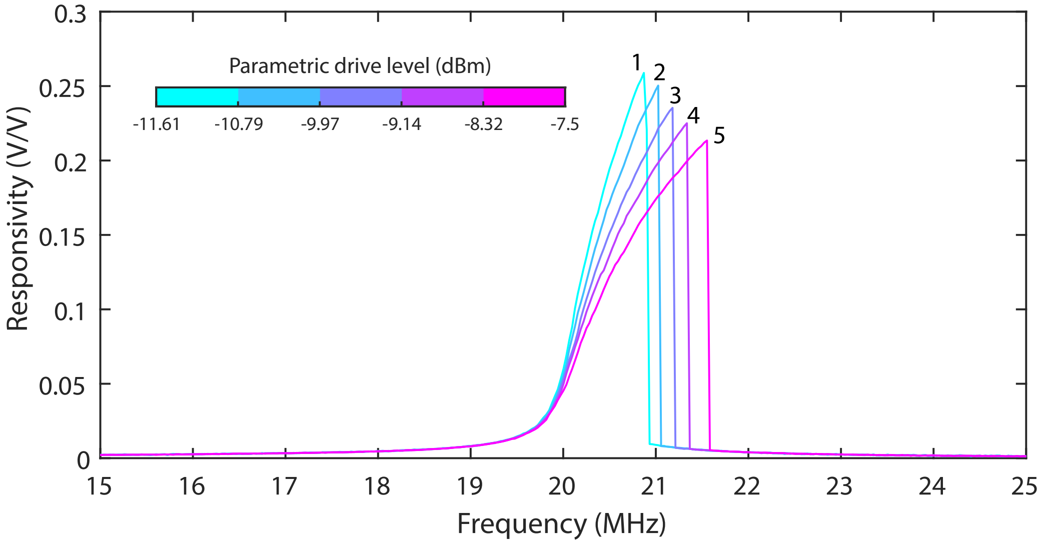

Supplementary Figure 7: **Responsivity measurements of the direct mode.** At higher drive levels decrease in responsivity is observed due to nonlinear damping.

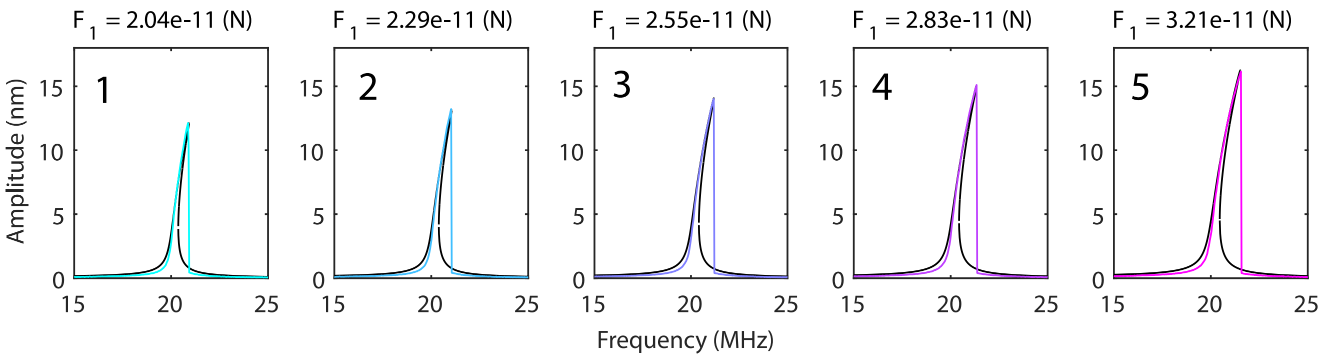

Supplementary Figure 8: **Model fits (black) to the experimental direct resonance curves from Supplementary Figure 7.** Model parameters from Table I have been used for all fits.

SUPPLEMENTARY NOTE VII. AMPLITUDE CALIBRATION

We measure the response of the graphene membrane in Volts from a Fabry–Pérot interferometer and convert the readings to nanometers using the nonlinear optical transduction technique presented in [4]. We note that for thin membranes and for high back-mirror reflectively, the reflected intensity $I(t)$ of the optical read-out can be

approximated by

$$I(t) = A + B\cos\left(4\pi\frac{g+\bar{x}(t)}{\lambda}\right), \tag{22}$$

where A and B are constants, $\bar{x}(t) = x\sin(\omega_F t)$ is the membrane displacement, g is the cavity depth, and λ is the wave-length of the light used for measurement. The reflected intensity $I(t)$ in Supplementary Equation (22) is a nonlinear function of the membrane displacement, and therefore, the read-out of a monochromatic signal will contain higher-order harmonics . The amplitude of these harmonics can be obtained from a Fourier expansion of the intensity $I(t) = \sum I_{n\omega_F}\sin(n\omega_F t)$, and harmonic balancing of Supplementary Equation (22). This will lead to the following relations for the first ($I_{1\omega_F}$) and the third ($I_{3\omega_F}$) harmonics of the motion [4]:

$$I_{1\omega_F} = -B\eta x\sin(\eta g) + \frac{1}{8}Bx^3\eta^3\sin(\eta g), \tag{23}$$

$$I_{3\omega_F} = -\frac{1}{24}Bx^3\eta^3\sin(\eta g), \tag{24}$$

in which $\eta = 4\pi/\lambda$. Taking the ratio of the two harmonics, one can find the motion amplitude as follows:

$$x = \frac{2\sqrt{6}}{\eta}\sqrt{\frac{I_{3\omega_F}/I_{1\omega_F}}{1+3I_{3\omega_F}/I_{1\omega_F}}}. \tag{25}$$

We can then obtain the linear transduction coefficient $C_{conv} = x/I_{1\omega_F}$ by averaging multiple data points on the nonlinear resonance curves in Supplementary Figure 9, in order to calibrate the response where $I_{3\omega_F}$ is below the noise level. The value we obtain for our experiments is $C_{conv} = 7.92\times10^{-7}$ (m/V). We also note that, above certain amplitudes, saturation of the resonance frequency curves become apparent for which the nonlinear transduction error becomes significant. We correct for this using [4]

$$x_{nl} = \left(1+\frac{1}{8}x^2\eta^2\right)x. \tag{26}$$

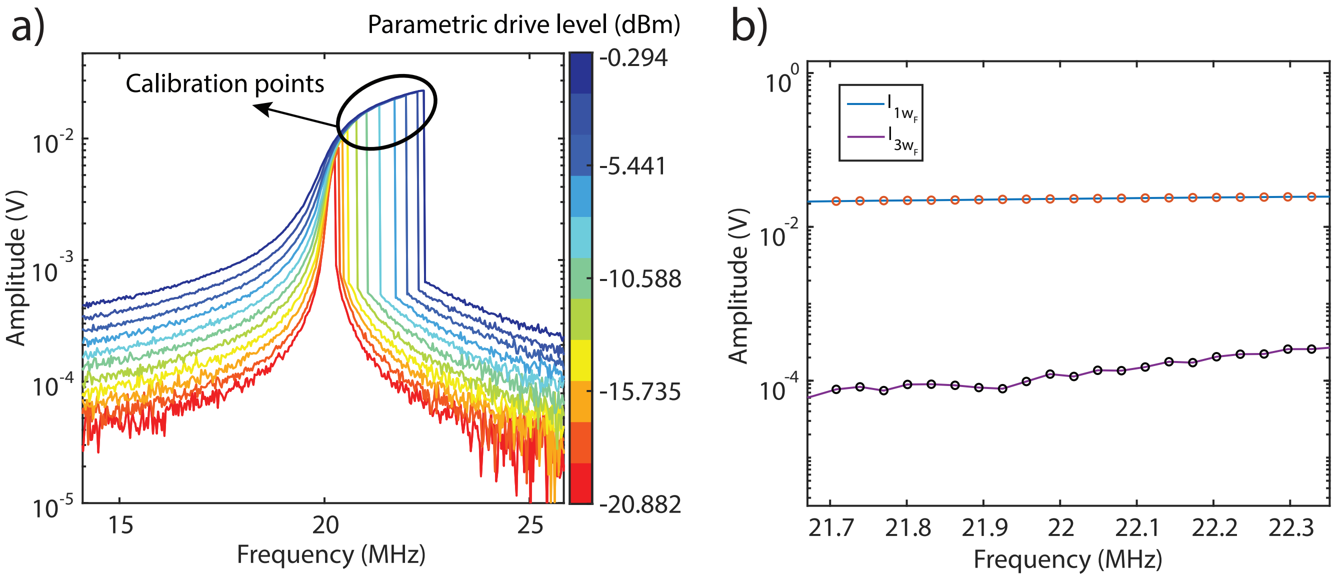

Supplementary Figure 9: **Calibration method.** (a) Nonlinear optical effect observed in the Duffing response has been used for amplitude calibration. (b) First and third harmonics arising from optical nonlinearity. The ratio of these harmonics are used to determine oscillation amplitude.

REFERENCES

[1] Lifshitz, R. & Cross, M. Nonlinear dynamics of nanomechanical and micromechanical resonators. *Review of nonlinear dynamics and complexity* **1**, 1–52 (2008).

[2] Amabili, M. *Nonlinear vibrations and stability of shells and plates* (Cambridge University Press, 2008).
[3] Guckenheimer, J. & Holmes, P. *Nonlinear Oscillations, Dynamical Systems, and Bifurcations of Vector Fields*. Applied Mathematical Sciences (Springer New York, 2013).
[4] Dolleman, R. J., Davidovikj, D., van der Zant, H. S. J. & Steeneken, P. G. Amplitude calibration of 2d mechanical resonators by nonlinear optical transduction. *Applied Physics Letters* **111**, 253104 (2017).
